# Exploring experiences and needs among children with cancer undergoing peripherally inserted central catheter insertion: A qualitative study

**DOI:** 10.1016/j.apjon.2025.100654

**Published:** 2025-01-09

**Authors:** Chengyang Li, Chunfeng Wang, Xueting Zhuang, Ying Wang, Yong Wu, Rong Hu

**Affiliations:** a The School of Nursing, Fujian Medical University, Fuzhou, China; b Department of Haematology, Fujian Medical University Union Hospital, Fuzhou, China; c College of Nursing, Fujian University of Traditional Chinese Medicine, Fuzhou, China

**Keywords:** Qualitative study, Children, Cancer, PICC, Experience

## Abstract

**Objective:**

This study aimed to explore the experiences of children with cancer undergoing peripherally inserted central catheter (PICC) insertion.

**Methods:**

This study employed a descriptive qualitative approach. A total of 20 children undergoing PICC insertion were enrolled through purposive sampling. Semi-structured interviews, supplemented by draw-and-tell techniques, were conducted to collect data. Thematic analysis method was employed to analyze the interview data.

**Results:**

Four themes and 11 subthemes were identified regarding the experience of peripherally inserted central catheter insertion in pediatric cancer patients, including (1) uncertainty (unknown procedure and lack of confidence); (2) high sensitivity (vulnerable to environmental influences and care what others think); (3) psychophysical changes (stress response, physical discomfort, disruption of daily routines, and accepted with pleasure); and (4) multidimensional needs (information, comfort, and self-actualization needs).

**Conclusions:**

The findings underscore the importance of gaining a deeper understanding of the symptoms and needs of pediatric cancer patients undergoing PICC insertion. By appreciating and honoring children’s voices, we can effectively cater to their distinct worries and guarantee they get the care and consideration they merit.

## Highlights

• This study identified four themes regarding PICC insertion for children with cancer: uncertainty, high sensitivity, psychophysical changes, and multidimensional needs.
• This study emphasizes child-centered care and identifies the unique issues and needs of pediatric patients during PICC insertion.
• This study could assist healthcare professionals in implementing targeted interventions to better manage the psychological and somatic experiences of children undergoing PICC insertion.

## Introduction

Childhood cancer is the sixth leading cause of the overall cancer burden worldwide, with an incidence rate of approximately 122.86 per million children in China.^1^^,^^2^ In recent years, advancements in treatment technology have led to a steady and significant increase in the cure rate for childhood cancer.^3^ Nonetheless, treating childhood cancer is a long process that requires extensive hospital stays, subjecting the child to invasive and uncomfortable physical and emotional treatments.^4^ It can be a challenging journey for both the child and their family.

Chemotherapy is one of the most effective treatments for childhood cancer.^2^ Peripherally inserted central catheters (PICCs) are a type of central venous catheter (CVC) inserted into peripheral veins (e.g., basilic, antecubital, or median cubital veins) and advanced to the cavoatrial junction or the lower third of the superior vena cava.^5^ PICCs are often used in patients who require long-term intravenous therapy, such as chemotherapy, antibiotics or total parenteral nutrition.^6^ The PICC offers numerous benefits, such as minimizing repeated punctures, decreasing vascular harm, ease of application, and cost efficiency, establishing it as a frequently utilized intravenous access for pediatric cancer patients undergoing chemotherapy.^7^, ^8^, ^9^

However, children with cancer often experience multiple potentially painful and distressing needle procedures, such as accessing the central venous port, lumbar puncture procedures or bone marrow punctures.^10^^,^^11^ Managing procedural pain in pediatric patients is one of the most challenging issues in hospital care.^12^ More than half of hospitalized children who underwent venipuncture experienced moderate to severe pain.^13^ These pain-related stresses not only affect physical, social, and cognitive functioning, but also impair the emotional and mental health of children.^14^ Children frequently struggle to deal with sickness and hospitalization due to unfamiliar people, unknown equipment, unpreparedness and lack of knowledge, leading to anxiety and uncertainty.^15^ Child-centered care implies that health professionals are obligated to ensure children’s rights and support their ability to express opinions on issues that impact their lives directly.^16^ It is crucial to understand children’s perspectives and experiences regarding their health to enhance support from health care providers for these children and others lacking coping strategies.^17^ By listening to children’s voices and incorporating their perspectives into medical care, health care providers can better support and comfort them during painful procedures.

PICC insertion involves a lengthy process with numerous steps and is more challenging than placing a regular peripheral venous catheter. Although studies have explored children’s procedural information needs and experiences, few have specifically addressed the PICC insertion process.^15^^,^^18^^,^^19^ Qualitative studies using in-depth interviews can enrich our understanding of children’s experiences with PICC insertion and ensure that children’s needs and preferences are considered in the development of effective symptom management strategies. Therefore, the purpose of this study was to collect qualitative data to understand the PICC insertion experience of children with cancer.

## Methods

### Study design

A descriptive qualitative study was conducted to explore the experience of PICC insertion in children with cancer. This study was reported following the Consolidated criteria for reporting qualitative research (COREQ) checklist (Supplementary Material 1).^20^^,^^21^

### Participants and setting

Participants were recruited through purposive sampling from a tertiary hospital with specialized pediatric oncology wards in Fujian Province, China. Participants were eligible for this study if they: (a) were aged between 5 and 12 years; (b) were diagnosed with cancer; and (c) were able to communicate in Chinese. Participants were excluded for this study if they: (a) had severe vital organ disease; (b) had mental or cognitive disorders.

### Data collection

Semi-structured interviews were conducted to understand children’s experiences of PICC insertion using draw-and-tell techniques. Draw-and-tell is an art-based technique that incorporates children’s drawings into the interview process, helping them access and express their sensory experiences.^22^ It is commonly used by psychologists and child life experts in research and clinical settings.^23^^,^^24^

The interview guide was developed by an expert panel consisting of a nursing professor, a qualitative nurse researcher, a psychological researcher, and a master’s candidate. The interviews were based on a semi-structured guide that covered five main areas: (1) mood before PICC insertion, (2) understanding of the PICC and the insertion process, (3) feelings and behaviors during insertion, (4) desired support, (5) postoperative effects and concerns, and (6) willingness to share their experiences. The individual interviews were conducted between November and December 2023. A pilot interview was conducted to ensure that children could easily understand the questions. All interviews were conducted by the first author (a Chinese registered nurse and nursing master’s candidate who had systematically learned qualitative research and child psychology courses) at the patients' bedsides. All of the interviews, which lasted between 15 and 35 min, were initiated with an opening question: “How are you feeling today?” Participants were encouraged to express their experiences freely. Probing questions such as “Why do you feel that way?” and “Can you elaborate?” were used to elicit further details. The first author was an intern in the pediatric ward where the study was conducted and had developed strong communication skills and a good rapport with the children.

Children under 8 years of age have better recall of events from today or yesterday,^25^ so we collected data within 24 hours of PICC insertion. Prior to the interview, children and their parents provided written consent and agreed to be audio recorded. Children were asked to draw their own PICC insertion experience based on the following question: “Can you draw the most impressive scene or feeling you had during the PICC insertion process?” After completing the drawing task, the researcher asked children to talk about their PICC insertion experience. To ensure the child’s privacy and minimize distractions, the bedside curtains were drawn during the interview to create a more confined environment. To meet ethical requirements, the parents of all participants were allowed to accompany them during the interview but were not permitted to influence the process. Interviews were conducted using child-friendly language and avoiding medical terminology. All interviews were recorded with a digital audio recorder and transcribed verbatim within 24 h of collection. The interviewer also captured facial expressions and gestures during the interviews. A total of 22 eligible children were invited to participate in the study, with two children declining due to feeling down. Data saturation was reached when no new meaning was obtained.

### Data analysis

Data collection and preliminary analysis were conducted simultaneously. Thematic analysis was used to analyze the interview data, with the following steps: i) Two authors transcribed the data and returned it to the children for verification and corrections; ii) Initial codes were generated from the transcripts after thorough review; iii) Similar codes were categorized into sub-themes; iv) Sub-themes were then reviewed and merged to create the overarching theme; v) Finally, all authors reviewed the themes together to ensure that the code in each theme was coherent and distinguishable. This approach was selected because it is generally used to provide in-depth explanations of children’s experiences.^26^ The data was transcribed and coded independently by two authors (CYL and CFW).

### Ethical considerations

Ethical approval for this study was obtained from the Ethics Committee on Biomedical Research of Fujian Medical University (IRB No. 2024/09). This study was conducted in accordance with the 1964 Helsinki Declaration and its later amendments or comparable ethical standards. Informed consent was obtained from each participant and their parent(s), who were also informed that they could withdraw from the study at any time. Participants' information was anonymized and kept confidential.

### Rigor

Several strategies were implemented to ensure the scientific rigor of the findings by guaranteeing credibility, confirmability, dependability, and transferability.^27^ To establish credibility, two researchers independently performed the data analysis, and discrepancies were resolved by discussion. To ensure confirmability, the researchers wrote reflective notes before and during the analysis process. To ensure dependability, the audio-recorded interviews were transcribed verbatim and verified with the participants. Transferability was ensured by applying specific selection criteria and including detailed demographic information.

## Results

### Characteristics of participants

Demographic and clinical characteristics of the participants are presented in Table 1. Ten boys and 10 girls (*n* ​= ​20), aged between 5 and 12 years (*M* ​= ​7.75) were included in this study, coding as P1∼P20. The majority of participants were non-local residents (*n* ​= ​14), diagnosed with leukemia (*n* ​= ​17), cared for by their mothers (*n* ​= ​18), and had been hospitalized within one to two weeks prior to the study (*n* ​= ​14).

**Table 1:** Participant demographic data (*N* ​= ​20).

Characteristics	*n* (%)/Mean
Age (years)	7.7
Sex	
Male	10 (50)
Female	10 (50)
Diagnosis	
Acute lymphoblastic leukemia	8 (40)
Acute myeloid leukemia	9 (45)
Hodgkin lymphoma	1 (5)
Non-Hodgkin’s malignant lymphoma	1 (5)
Hepatoblastoma	1 (5)
Residence	
Local	6 (30)
Non-local	14 (70)
Household size	
1–3	7 (35)
4–5	6 (30)
> 5	7 (35)
Caregivers	
Father	3 (15)
Mother	17 (85)
Time since diagnosis (week)	
< 1	3 (15)
1–2	14 (60)
> 2	3 (15)
First PICC insertion	
Yes	18 (90)
No	2 (10)

PICC, peripherally inserted central catheter.

### Themes

Four themes and 11 subthemes reflecting the experience of PICC insertion in children with cancer were identified, including uncertainty, high sensitivity, psychophysical changes, and multidimensional needs. The detailed results are summarised in Table 2.

**Table 2:** Summary of themes and sub-themes.

Themes	Sub-themes
**Uncertainty**	Unknown procedure
	Lack of confidence
**High sensitivity**	Vulnerable to environmental influences
	Care what others think
**Psychophysical changes**	Stress response
	Physical discomfort
	Disruption of daily routines
	Accepted with pleasure
**Multidimensional needs**	Information needs
	Comfort needs
	Self-actualisation needs

### Theme 1: uncertainty

A common issue that exists in pediatric patients undergoing PICC insertion is child stress. This stress includes uncertainty about the procedure and lack of confidence . They may feel uncertainty when faced with these stress.

#### Unknown procedure

Most of the children were undergoing PICC insertion for the first time and lacked proper health education, leading to insufficient understanding of the PICC. They tend to predict how new needle procedures will affect them based on past experiences.

*“I never feared injections until after the bone marrow aspiration. The bone marrow aspiration was so excruciating. So I’m unsure of what to expect with this PICC insertion. I’m worried it will be as painful as the bone marrow aspiration, and I hope the needle is not too thick.”(P13)*

*“Lying in bed, I felt that time was passing so slowly and I couldn’t see what was going on, I just kept asking the nurse why it wasn’t done yet. This is a torment for me, soon to be impatient, because the nurse gave me an indwelling needle before and it was over quickly.” (P18)*

#### Lack of confidence

The children were not confident about living with a PICC after discharge and feared they would not be able to manage potential complications. In addition, they have concerns about the successful treatment of their disease.

*“Due to recent bleeding in the area, I am worried that it may persist after leaving the hospital. I am also worried that if I accidently touch it post-discharge, it could start bleeding again, and I am unsure of what to do if that happens without any medical staff available to assist me.” (P16)*

*“The nurse said that this PICC can stay in the arm for a year, and I don’t know how long I’ll need to be treated for this disease or if I’ll ever be cured.” (P11)*

### Theme 2: high sensitivity

Heightened sensory perception allows children to perceive subtle environmental and emotional changes, which may lead to both beneficial and detrimental outcomes. This sensitivity enables a more profound experience of the world, but also increases vulnerability to stress and anxiety. Sometimes children are sensitive to changes in their parents' moods and can put themselves in their parents' shoes. They also care about what others think of them, which indicates that children have developed a certain degree of social awareness and self-knowledge.

#### Vulnerable to environmental influences

The unfamiliar environment of the operating room can have a psychological impact on children. Cool temperatures, distressed children, and health care professionals in sterile gowns and masks can overwhelm and frighten young patients. Additionally, the smell of sterilized water also affects the experience of the child. Some children are not accustomed to the smell of disinfectant in hospitals, which makes them uncomfortable. They may even have a physical reaction to it.

**Fig. 1:**
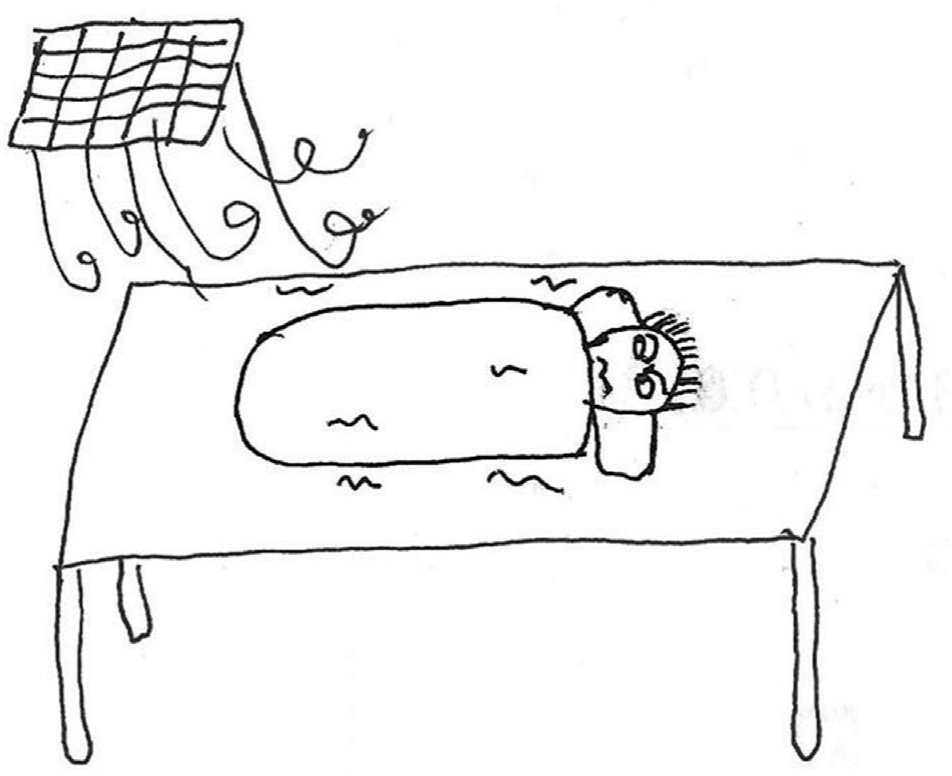
“*I am shivering from the cold* …” (P6).

*“The room was eerie and cool, giving me a slight feeling of fear. They provided me with a small quilt, yet I still felt cold.” (P6) (*Fig. 1*)*

*“While waiting outside the operating room I heard a child crying inside and it made me feel scared.”(P12)*

*“The smell of disinfectant in operating room was so strong and pungent (frowning). I’m very sensitive to the smell of disinfectant and I want to vomit when I smell it.” (P14)*

#### Care what others think

**Fig. 2:**
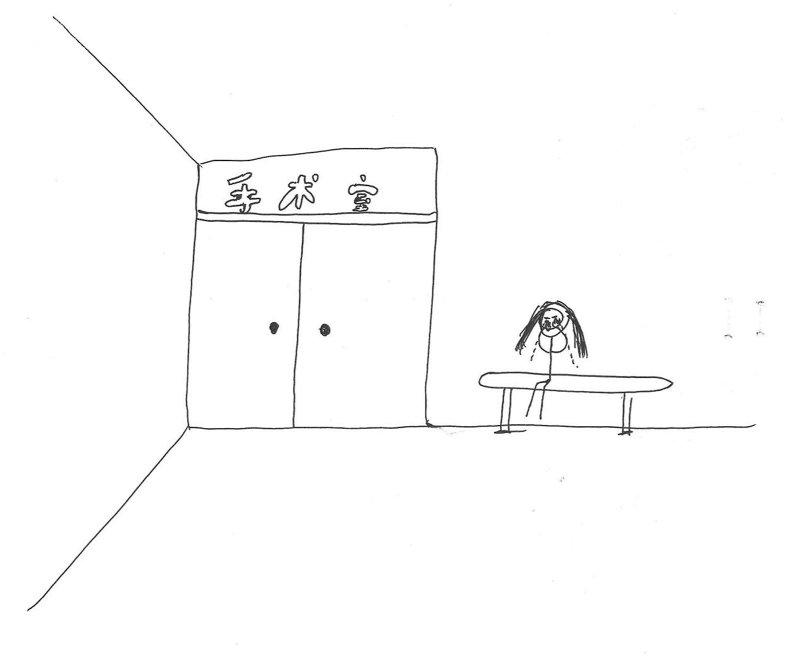
“*Crying mom outside the operating room*” (P8).

Children also care about what others think, which shows that children have developed a certain degree of social awareness and self-knowledge. Sometimes children are sensitive to changes in their parents' moods and can put themselves in their parents' shoes. A 10-year-old boy’s (P8) expressed concern about his mom’s emotional state. This empathy is vividly depicted in his drawings, a scene imagined while he was in the operating room (Fig. 2). Some children tend to cry during their PICC insertion and do not want others to see their embarrassment. Surprisingly, some children consider it a stigma to live with tubes and are reluctant to show them to others after discharge.

*“I entered the operating room alone, and concerned that my mother would be anxious outside. She felt uneasy and teared up each time I got an injection.” (P8)*

*“I went into the operating room alone because I didn’t want my mom and dad to come with me. They always laughed at me when I cried during my injections (pouting).” (P2)*

*“If I go to school in the future with this tube on my arm, I’m afraid other students will see it and might not want to play with me if they know I’m sick.”(P16)*

### Theme 3: psychophysical changes

Psychophysical changes refer to the interplay between emotional and physical reactions to the PICC insertion process. Children often exhibit increased anxiety levels, which can lead to physical symptoms such as sweating, heart palpitations, and muscle tension. Children experienced stress response, physical discomfort and disruption of daily routines during PICC insertion. On the positive side, the procedure can change their acceptance of PICC.

#### Stress response

Although some children are emotionally stable prior to the procedure, they still experience significant stress when the needle is administered. While being aware of the impending pain, they will rationally remain cooperative and understand the potential consequences due to their body’s fidgeting. Children may become tense when someone else touches their newly placed PICC, concerned about potential displacement or pain.

*“When the nurse mentioned that I needed an anesthetic shot, I felt very nervous and wanted to pee. My hands were clenched, my palms were sweating, my heart was racing.” (P10)*

*“I lay on the bed, clenched my teeth slightly, and remained completely still. If I move, they might thread the needle crookedly.” (P16)*

*“After the PICC, I get nervous when someone touches it and my muscles tense up.”(P14)*

#### Physical discomfort

The discomfort associated with a child’s PICC insertion experience includes pain from anesthesia and postoperative activities. All children reported experiencing pain, primarily from anesthesia and postoperative activities. While children acknowledge the pain-relieving benefits of anesthesia, they perceive the process as painful for them as well. In addition, postoperative pain can limit their daily activities. Some children feel a foreign body sensation in their arms, causing them to not be able to move as they normally do. After the procedure, some children are allergic to the protective dressing of the PICC and may experience an itchy sensation.

*“At the start of the anesthesia, I experienced a sudden surge of pain which quickly subsided. I have a low tolerance for pain and tend to cry easily.” (P8)*

*“I always feel like there’s something in my arm. It’s a strange feeling. My dad bought me Legos and I want to play with them, but I’m afraid to move my arm.” (P3)*

*“This tape is a little itchy on my arm and sometimes I can’t help but touch it. My mom sometimes rubs it gently for me.” (P5)*

#### Disruption of daily routines

Children find it quite inconvenient to have a PICC tube on their arm in their daily lives. Given their current inability to bathe conventionally, washing their bodies in bed may lead to discomfort. In addition, the placement of a PICC may interfere with the quality of the child’s sleep, preventing them from sleeping peacefully.

*“I can’t take a bath as usual. My mom just wipes my body with a wet towel. It feels uncomfortable to shower like this than before because of the sticky body.” (P2)*

*“It’s not very convenient to hold a pen to write with. I have to be careful not to bend my arm too much, especially during infusions, to make sure the medicine flows smoothly without kinks in the tube.” (P4)*

*“It affects my sleep, and I don’t sleep as freely as I used to. The nurse told me not to press on it when I was asleep, and then I didn’t dare to turn over.” (P10)*

#### Accepted with pleasure

Through health education by health care professionals or parents and hands-on experience, children learn that having a PICC placed can enhance their healing experience. Because of the advantage of being able to reduce the number of injections they have to take, they are able to readily accept the PICC.

*“The most significant change is that I no longer need the same type of indwelling needle I used before (smiling). I’ve used this type of indwelling needle several times and it stops working after a few days. The PICC could stay in my arm for a long time.” (P2)*

*“I didn’t know the purpose for the PICC line before, but now I understand it will lead to less pain and faster recovery.” (P6)*

### Theme 4: multidimensional needs

This theme discusses the diverse needs of children, such as informational, comfort, and self-actualization needs. The multifaceted needs of children in health care go beyond physical health to encompass their emotional, psychological, and social well-being.

#### Information needs

Many children are curious about the PICC insertion process before the procedure. However, interviews revealed that health care professionals often do not provide children with details about the procedure. Some children may not be able to understand what the health care provider is saying and would like to be taught in a simple way. During PICC insertion, a child’s curiosity may manifest itself in an interest in the medical device, the procedure, or the behavior of the medical staff.

*“I wish the nurse would talk to me about some of the PICC information, but they often talk to mom and dad. And I especially wanted to know if the pain was intense during the injections.” (P11)*

**Fig. 3:**
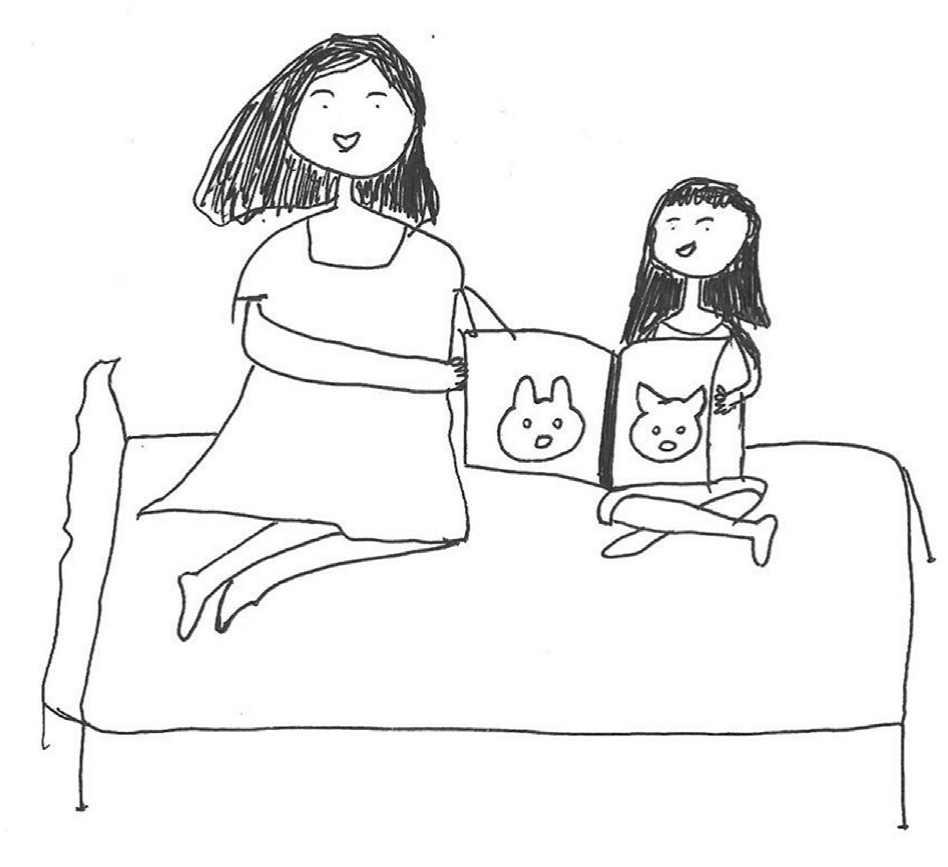
“*My mom and I are reading a picture book about PICC*” (P14).

*“I couldn’t understand the video they showed me. I enjoy reading picture books, and I have one here about hospitalization. If I had a picture book about PICC insertion to read before the procedure, I might not have been as afraid to cry.” (P14) (*Fig. 3*)*

*“While lying in bed, I asked the nurse’s sister to bring me the PICC to see what it looked like.” (P7)*

#### Comfort needs

Most children want their parents to be there for them, which would help calm their nervousness. During the procedure, many children expressed a need for ongoing communication during the procedure, where they desired reassurance and updates on the progress of the procedure. This not only helped reduce anxiety but also fostered a sense of control and participation in the treatment process.

*“I was chatting with the nurse so I wasn’t as nervous. I wanted them to tell me what they were doing, what they were doing next, and if they were almost done.” (P4)*

**Fig. 4:**
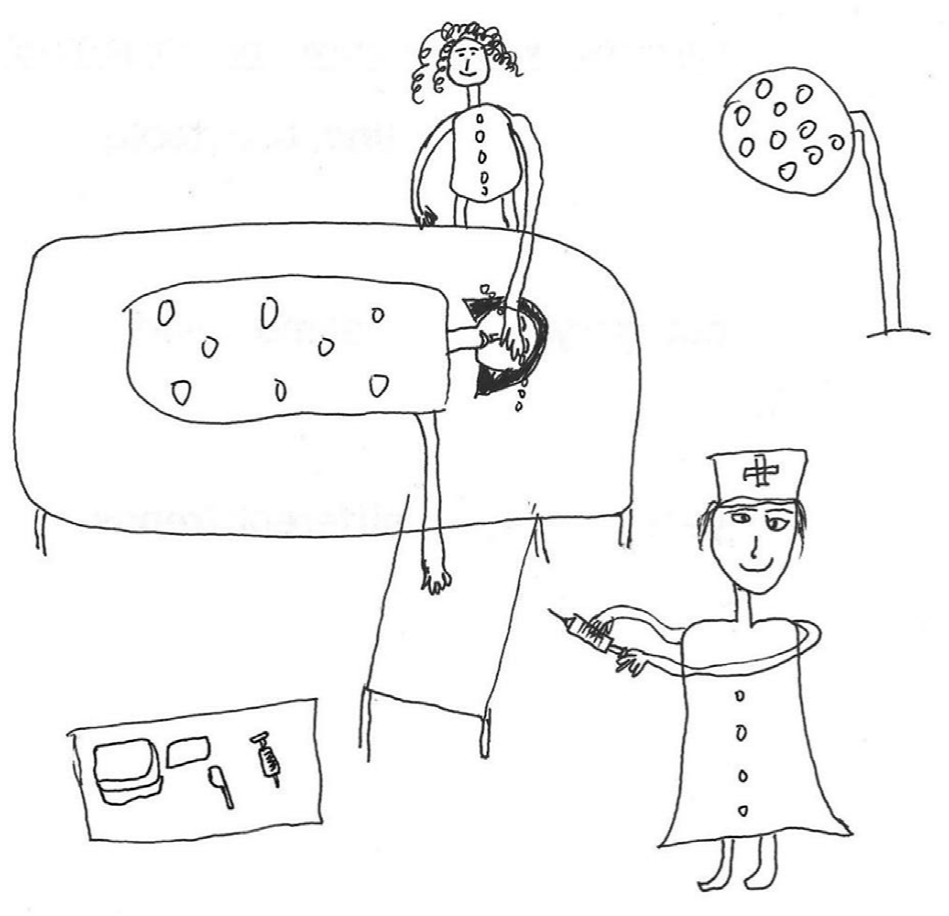
“*Mom covered my eyes*” (P13).

*“I cried all the time. When I had to get a needle, I had to get my mom to cover my eyes before I dared. I wanted my mom by my side, but the nurse told them to wait outside.” (P13) (*Fig. 4*)*

#### Self-actualization needs

We also found that some children have a need for self-actualization. They wanted to be rewarded by their parents. Some children expressed a desire for self-actualization, often by sharing their experiences with peers. This not only provided a sense of personal achievement but also allowed them to feel that they were helping others facing similar challenges. This behavior aligns with Maslow’s theory of self-actualization, where individuals seek fulfillment by contributing to others' well-being.

**Fig. 5:**
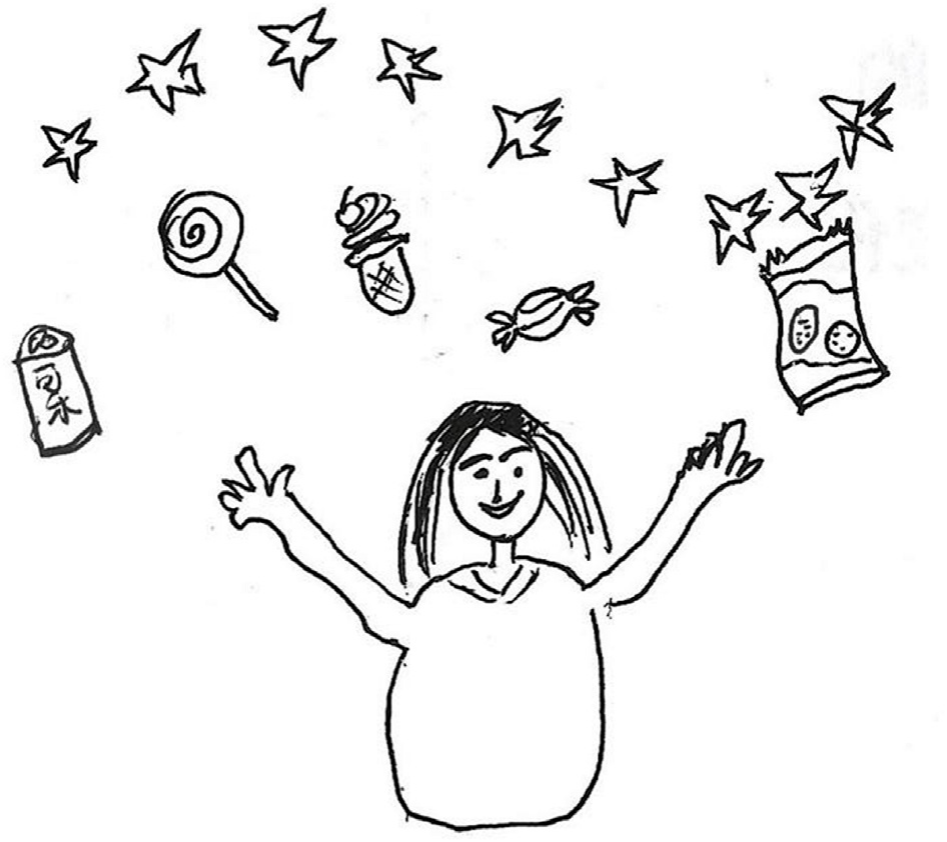
“*Mom rewarded me with lots of snacks* …” (P15).

*“My mom usually gives me stars that I can redeem for prizes, such as my favorite snacks and toys. She mentioned that I could earn 20 stars for performing well during this procedure.” (P15) (*Fig. 5*)*

*“Sharing my experience can encourage them feel more at ease, and bring me joy. My mom’s colleague’s daughter, who also has leukemia, shared her experience with me, which I found helpful.” (P17)*

## Discussion

This is the first qualitative study of children’s perceptions of PICC insertion. It provides a comprehensive exploration of the experiences of children with cancer undergoing PICC insertion. The findings reveal four main themes: uncertainty, high sensitivity, psychophysical changes, and multidimensional needs. These themes encapsulate the physical, emotional, and psychological challenges faced by pediatric patients during PICC insertion and highlight the importance of a holistic approach to their care.

Uncertainty is strongly linked to anxiety, depression, and overall psychological symptoms (e.g., psychosis, somatic, and obsessive-compulsive symptoms), and negatively correlated with social support.^28^ Uncertainty is associated with cognitive coping and relies on cognitive schemas or structures for guidance.^29^ When children lack these cognitive frameworks to understand illness treatment events, uncertainty increases, leading to greater psychological distress and reduced quality of life.^30^ This uncertainty was further exacerbated by their past experiences with painful procedures, leading to anxiety and fear. To effectively address this issue, health care providers must recognize the importance of a comprehensive educational program that is specifically designed to cater to the cognitive and emotional needs of pediatric patients. This indicates that health care providers can implement a structured educational program that includes visual aids, interactive sessions, and age-appropriate language to demystify the PICC process, potentially enhancing their confidence during PICC insertion. Additionally, we can incorporate psychological support strategies, such as clown therapy,^31^ distraction technique,^32^ and parental presence,^33^ to create a more supportive environment. This not only benefits the child in the immediate context of the procedure but also potentially reduces long-term anxiety and fosters a more collaborative relationship with the health care system.

While uncertainty primarily reflects a lack of knowledge about the procedure, high sensitivity highlights how children’s emotional responses to their surroundings and the actions of caregivers exacerbate their stress levels during PICC insertion. The theme of high sensitivity underscores the children’s acute awareness of their environment and the emotions of those around them. The sterile and often cold operating room environment, the smell of disinfectants, and the sight of health care professionals in masks and gowns can be overwhelming. These factors can heighten the children’s stress and anxiety. Therefore, creating a child-friendly environment that minimizes these stressors is essential. Some children care what others think, revealing the complex social awareness and self-consciousness that children with cancer experience during their treatment. Children’s concerns about what others think extend beyond their immediate treatment environment. They are aware of others' judgments and reactions to their condition and the visible signs of their treatment, such as the PICC line. This awareness can lead to feelings of embarrassment, stigma, and a desire to hide their condition from others. For example, some children expressed anxiety about returning to school with a PICC line, fearing that their peers would react negatively or exclude them because of their illness or the presence of the catheter. This sensitivity to social perceptions underscores the importance of psychological support and counseling for children with cancer. Health care providers can play an important role in alleviating these concerns by normalizing the experience of having a PICC line, emphasizing its temporary nature, and addressing common misconceptions that children may have about how others will perceive them. The empathic sensitivity of children with cancer to their parents' emotional states is a reminder of the deep empathic connections that exist in families experiencing the challenges of childhood cancer.^34^ This sensitivity reflects children’s emotional maturity and innate ability to perceive and respond to their caregivers' emotional cues. Children’s ability to empathize with their parents suggests that they are not only able to cope with their own fears and anxieties, but also empathize with their family’s emotional burdens. This two-way emotional communication underscores the importance of family-centeredness in pediatric oncology care. Providers must recognize that the emotional health of parents and caregivers is closely linked to the emotional health of the child.^35^ Helping parents manage their own emotions, in turn, creates a more positive and supportive environment for the child.

Children experienced significant psychophysical changes, including stress response, physical discomfort, and disruption of daily routines. Even emotionally stable children experienced discomfort during the PICC line insertion procedure and postoperatively. The most distressing of these symptoms is pain, which, if not adequately managed, can lead to long-term consequences such as increased anxiety and non-compliance with care.^36^ This highlights the critical need for medical personnel to be well-versed in an array of pharmacological and non-pharmacological pain management techniques, to ensure that children are provided with the most effective relief from pain and discomfort.^11^ This may include incorporating age-appropriate coping strategies, encouraging play and social interaction, and working closely with the family to ensure a smooth transition back to normal activities, with the family empowered to advocate for the child’s comfort and participation. Some children fear moving due to pain, which can impact blood flow and increase thrombosis risk.^37^ Postoperative education should be a priority, with a focus on reassuring children that normal movement of the limb is not only safe but encouraged. This can be achieved through clear communication about the benefits of gentle movement and the potential risks associated with immobility. Additionally, children should be counseled against behaviors such as excessive rubbing or scratching around the PICC site to prevent skin irritation or infection. Foreign body sensation is a common discomfort for patients, which may be caused by the presence of the catheter, positional movement, or skin sensitivity to the catheter material. Implants may cause emotional distress, anxiety, and depression in some patients, influenced by individual differences and sensitivity to symptoms.^38^ These somatic discomforts can lead to increased anxiety and fear, making the experience even more challenging for both the children and health care providers. Therefore, medical staff should assist and motivate patients to enhance their capacities to deal with disease cognition and behaviors. By eliminating the need for repeated needle sticks in peripheral veins, PICC lines can minimize the pain and distress associated with frequent venipuncture. This is especially beneficial for children who have a low tolerance for pain or who are sensitive to repeated invasive procedures. Children’s understanding of the purpose and benefits of a PICC line can lead to acceptance and a positive attitude toward the device as a way to improve their health and well-being. The presence of a PICC line can be an inconvenience in a child’s daily routine. Activities such as learning, bathing and sleeping may require adjustments to prevent complications such as infection, dislodgement or damage to the catheter. We can provide clear instructions on how to safely perform daily activities, such as bathing with a waterproof dressing or adjusting sleeping positions to protect the catheter site.

We found that children have multidimensional needs that need to be met. Child-centered care (CCC) in health care recognizes the rights of children and emphasizes their independence and involvement in the communication process.^39^ Despite the recognized benefits of CCC, the reality is that children often lack sufficient information during hospitalization and treatment. The tendency of physicians to direct preoperative information solely to parents or guardians.^40^ In addition, most parents prefer not to share information about surgery and anesthesia with their children because they are concerned that it may cause anxiety and stress.^41^ The lack of appropriate information provided by health care professionals can exacerbate children’s feelings of uncertainty and anxiety, which is further compounded by their limited understanding of medical terminology. This study found that children expressed a desire for engaging and interactive health education that addressed their specific needs and concerns. By using age-appropriate resources such as picture books and storytelling, children can gain a better understanding of medical procedures, including what to expect and how to prepare, which aligns with the recommendations of Shave et al.^42^ The psychological state of children must be considered by health care workers and caregivers. Providing comfort through verbal and physical reassurance can significantly alleviate anxiety, especially during invasive procedures like injections. Recognizing and supporting children’s desire for self-actualization is also crucial; children who feel they can contribute and be recognized for their bravery can experience a sense of pride and accomplishment.

This study highlights the significant psychological, emotional, and informational needs of children with cancer who are treated with PICC intubation. It has been shown that addressing these needs through child-centered care, improved communication, psychosocial support, and family involvement can facilitate pediatric cancer care.^43^^,^^44^ These supports can reduce anxiety, enhance coping strategies, increase treatment adherence and improve the overall well-being of children and their families. In the long term, health care providers will improve the quality of comprehensive care by incorporating these findings into clinical practice and developing targeted symptom management strategies tailored to children.

### Implications for nursing practice and research

This research offers critical understanding of the unique experiences and needs of children with cancer undergoing PICC insertion. It emphasizes the critical role of health care professionals in understanding and responding to the emotional and informational requirements of pediatric patients to facilitate appropriate interventions. Health care providers should recognize the importance of a positive patient experience and integrate these findings to improve future clinical practices and outcomes for children undergoing medical procedures. The findings of this study, although based on a sample from southeastern China, have international relevance in the context of pediatric cancer care. By emphasizing a child-centered approach that prioritizes emotional and psychological needs, the study’s conclusions can be adapted to different cultural and health care settings. Globally, pediatric oncology units can benefit from incorporating age-appropriate education, psychological support, and family-centered care strategies that address the universal challenges children face during invasive procedures.

### Limitations

We acknowledge some limitations of this study. First, participants were chosen from a hospital in southeastern China, which could impact the generalizability of the results. Second, the recruitment of participants was limited by age, so the findings may not be applicable to children of all age groups. Third, there is a possibility of subjective bias in this study due to the interviewer, despite our efforts in quality control. Lastly, as children mature, their cognitive, linguistic, and logical reasoning abilities progressively refine. These developmental nuances can shape the way youngsters across various ages and cognitive stages interpret and articulate their responses in interviews, thereby affecting the richness and profundity of the dialogue.

## Conclusions

In our study, children experienced uncertainty, high sensitivity, psychophysical changes, and multidimensional needs. They faced both physical and emotional challenges and often lacked adequate medical attention. Recognizing and addressing these issues promptly is crucial for health care professionals to provide effective intervention and understand the child’s perspective. Enhancing this understanding can lead to improved outcomes for both health care and the child’s well-being.

## CRediT authorship contribution statement

Chengyang Li: Conceptualization, Formal analysis, Data curation, Investigation, Writing – original draft. Chunfeng Wang: Formal analysis, Data curation, Writing – review & editing. Xueting Zhuang: Data curation, Writing – review & editing. Ying Wang:Writing – review & editing. Yong Wu: Resources, Project administration. Rong Hu: Methodology, Supervision, Writing – review & editing. All authors had full access to all the data in the study, and the corresponding author had final responsibility for the decision to submit for publication. The corresponding author attests that all listed authors meet authorship criteria and that no others meeting the criteria have been omitted.

## Ethics statement

The study was approved by the Ethics Committee on Biomedical Research of Fujian Medical University (IRB No. 2024/09). All participants provided written informed consent.

## Funding

This study received no external funding.

## Data availability statement

Data supporting the findings of this study are available upon reasonable request from the corresponding authors, YW and YW. These data will not be made public owing to privacy and ethical restrictions.

## Declaration of generative AI and AI-assisted technologies in the writing process

No AI tools/services were used during the preparation of this work.

## Declaration of competing interest

The authors declare that there is no conflict of interest. One of the correspongding authors, Dr. Ying Wang, is an editorial board member of *Asia–Pacific Journal of Oncology Nursing*. The article was subject to the journal’s standard procedures, with peer review handled independently of Dr. Wang and their research groups.
