# Supplementary material for: Exploring experiences and needs among children with cancer undergoing peripherally inserted central catheter insertion: A qualitative study
Source: Asia Pac J Oncol Nurs. 2025 Jan 9;12:100654. doi: 10.1016/j.apjon.2025.100654 (PMC11800096; doi:10.1016/j.apjon.2025.100654)
Supplement: Multimedia component 1 [file mmc1.docx]

**Consolidated criteria for reporting qualitative studies (COREQ): 32-item checklist**

Developed from:

Tong, A., Sainsbury, P., & Craig, J. (2007). Consolidated criteria for reporting qualitative research (COREQ): a 32-item checklist for interviews and focus groups. International Journal for Quality in Health Care, 19(6), 349-357.

| **No. Item** | **Guide questions/description** | **Reported on Page #** |
| --- | --- | --- |
| **Domain 1: Research team and reﬂexivity** | | |
| ***Personal Characteristics*** | | |
| 1. Inter viewer/facilitator | Which author/s conducted the interview or focus group? | Page 5-7: Data collection  The first author. |
| 2. Credentials | What were the researcher’s credentials? E.g. PhD, MD | Title page |
| 3. Occupation | What was their occupation at the time of the study? | Master’s degree student majoring in nursing, PhD, Chief Physician, and Professor. |
| 4. Gender | Was the researcher male or female? | Three female and two male. The first author is male. |
| 5. Experience and training | What experience or training did the researcher have? | Page 5-7: Data collection |
| ***Relationship with participants*** | | |
| 6. Relationship established | Was a relationship established prior to study commencement? | Page 5-7: Data collection  Yes, to acquire the most accurate information, the interviewer developed a trusting relationship with the participants by taking part in nursing activities as a nurse, before data collection. |
| 7. Participant knowledge of the interviewer | What did the participants know about the researcher? e.g. personal goals, reasons for doing the research | Page 4-5: Setting and Participants |
| 8. Interviewer characteristics | What characteristics were reported about the inter viewer/facilitator? e.g. Bias, assumptions, reasons and interests in the research topic | Page 5-7: Data collection |
| **Domain 2: study design** | | |
| ***Theoretical framework*** | | |
| 9. Methodological orientation and Theory | What methodological orientation was stated to underpin the study? e.g. grounded theory, discourse analysis, ethnography, phenomenology, content analysis | Page 4: Study Design |
| ***Participant selection*** | | |
| 10. Sampling | How were participants selected? e.g. purposive, convenience, consecutive, snowball | Page 4-5: Setting and Participants  Purposive sampling. |
| 11. Method of approach | How were participants approached? e.g. face-to-face, telephone, mail, email | Page 4: Study Design  Face-to-face. |
| 12. Sample size | How many participants were in the study? | Page 4-5: Setting and Participants |
| 13. Non-participation | How many people refused to participate or dropped out? Reasons? | Page 4-5: Setting and Participants & Table 1 |
| ***Setting*** | | |
| 14. Setting of data collection | Where was the data collected? e.g. home, clinic, workplace | Page 5-7: Data collection |
| 15. Presence of non-participants | Was anyone else present besides the participants and researchers? | Parents |
| 16. Description of sample | What are the important characteristics of the sample? e.g. demographic data, date | Table 1 |
| ***Data collection*** | | |
| 17. Interview guide | Were questions, prompts, guides provided by the authors? Was it pilot tested? | Page 5-7: Data collection  Yes. |
| 18. Repeat interviews | Were repeat interviews carried out? If yes, how many? | Page 5-7: Data collection  No. |
| 19. Audio/visual recording | Did the research use audio or visual recording to collect the data? | Page 5-7: Data collection  Yes. |
| 20. Field notes | Were ﬁeld notes made during and/or after the interview or focus group? | Page 5-7: Data collection  Yes. |
| 21. Duration | What was the duration of the interviews or focus group? | Page 5-7: Data collection  Yes. |
| 22. Data saturation | Was data saturation discussed? | Page 4-5: Setting and Participants |
| 23. Transcripts returned | Were transcripts returned to participants for comment and/or correction? | Page 7: Data Analysis  Yes. |
| **Domain 3: analysis and ﬁndings** | | |
| ***Data analysis*** | | |
| 24. Number of data coders | How many data coders coded the data? | Page 7: Data analysis |
| 25. Description of the coding tree | Did authors provide a description of the coding tree? | No. |
| 26. Derivation of themes | Were themes identiﬁed in advance or derived from the data? | Page 7: Data analysis  Derived from the data. |
| 27. Software | What software, if applicable, was used to manage the data? | No. |
| 28. Participant checking | Did participants provide feedback on the ﬁndings? | No. |
| ***Reporting*** | | |
| 29. Quotations presented | Were participant quotations presented to illustrate the themes/ﬁndings? Was each quotation identiﬁed? e.g. participant number | Page 8-16: Results.  Yes. |
| 30. Data and ﬁndings consistent | Was there consistency between the data presented and the ﬁndings? | Yes, all the findings were consistent with the data. |
| 31. Clarity of major themes | Were major themes clearly presented in the ﬁndings? | Page 8-16: Results. Table 2.  Yes. |
| 32. Clarity of minor themes | Is there a description of diverse cases or discussion of minor themes? | Page 8-16: Results. Table 2.  Yes. |
